# Book Reviews

**Published:** 1852-11

**Authors:** 


*Operative Surgery, illustrated. Containing more than Nineteen Hundred Engravings, including Two Hundred Original, and Fifty Colored Drawings, with Explanatory Text.* By R. U. Piper, M. D. *Also a Chapter on the use of Ether in Surgery.* By H. I. Bigelow, M. D., Prof. of Surgery in Harvard University. Boston: Ticknor, Reed & Fields. 1852. 12mo, pp. 384.

The plan and general scope of this work are set forth in the following extract from the preface: "A picture often presents to the eye, at a glance, more satisfactory and precise information than a protracted description. With this belief, and with a desire to make existing knowledge available and cheap, rather than to enlarge its boundaries, the present work was undertaken. It is not designed to be a methodical treatise, but rather a general storehouse of matters connected with practical surgery; and although it really contains ample illustrations of all the regular operations, both of the theatre and the dissecting room, the surgeon will also find here a large amount of useful and practical information, upon a wide range of collateral subjects connected with surgical manipulation."

The work seems to us to be one which will prove highly acceptable to medical students, and to practitioners who are called on (as most practitioners are) to make surgical operations. It is filled with beautiful engravings representing every variety of surgical manipulation, instruments, bandaging, etc. As the author remarks in the quotation just given, a picture often conveys a more correct idea, and more quickly, than verbal descriptions, however vivid. Moreover, in this work is furnished, at a small price, a host of illustrations selected from numerous treatises which, from their cost, are not within the reach of a majority of either students or practitioners. The getting up of the work is admirable, being in the best style of Boston typography. The author, Dr. Piper, has been at great pains in its preparation, and we hope his labors will be rewarded by a prompt and extensive demand for it.

---

*Practical Chemistry, a branch of Medical Education; considered in a brief letter to his class.* By Alfred L. Kennedy, M. D., Lecturer in the Philadelphia School of Chemistry, etc., etc.

The object of this letter is to advocate the importance of a greater relative attention to practical chemistry in medical schools. The writer thinks that the medical student should devote a portion of the period of his attendance at medical lectures, to *working* in the laboratory under the direction of a "demonstrator in chemistry." There can be no question as to the superior value of this plan of instruction, nor as respects the advantage of giving more attention to chemistry than is generally done by medical students. It is clear that from organic or animal chemistry we are to look for many of the important revelations which are hereafter to enrich medical science. Investigations hitherto, for the most part, have related to the *effects* of disease — the changes produced in the solid structures. With the revival of humoralism (which is mainly owing to the labors of the chemist,) and the progress of analytical researches pertaining to the fluids in health and disease, we may hope to attain to a better knowledge of the intimate nature of the relations of morbid actions. There iscertainly no province of medicine which promises richer fruits than this to reward the efforts of genius and industry.

*The Physician's Visiting List, Diary, and Book of Engagements, for* 1852. Philadelphia: Lindsay & Blakiston, 25 South Sixth-street, above Chestnut.

The idea of a book for the convenience of physicians in noting their daily visiting list, engagements, etc., is an excellent one. The plan of that issued by Lindsay & Blakiston, however, is susceptible of improvement. The spaces for noting daily visits are too small. It is often desirable to state the person visited and the nature of the service rendered. The copy which we have used for the past year, allowing twenty-five patients per week, is too restricted. We reckon there are few city practitioners in extensive practice, who do not have to make charges against more than this number. It strikes us that it would be better to appropriate a page to each day. This would leave room for everything relating to the business of the day which it would be desirable to note. To render the book less bulky, a considerable portion of the extra pages might be dispensed with. At present, we know no better, nor indeed any other book than this designed for the same object, and we accordingly commend it to the favor of our readers. It contains an almanac for the year; a posological table; a list of poisons and their antidotes; and an abstract of the proceedings of the last meeting of the American Medical Association. These will be found useful for reference.

